# Periodontitis Severity Grading Scale and C-Reactive Protein: A Possible Relation

**DOI:** 10.7759/cureus.41618

**Published:** 2023-07-10

**Authors:** Jasuma Rai, Vandana Shah, Monali Shah

**Affiliations:** 1 Department of Periodontology, K M Shah Dental College & Hospital/Sumandeep Vidyapeeth, Vadodara, IND; 2 Department of Oral and Maxillofacial Pathology, K M Shah Dental College & Hospital, Vadodara, IND

**Keywords:** systemic inflammation, suppuration, periodontitis, c reactive protein, bleeding on probing

## Abstract

Background

C-reactive protein (CRP) is the first acute-phase protein and is an exceptional investigative marker for local and systemic inflammation. The periodontitis severity grading scale (PSGS) indicates the extent of periodontal inflammation. Therefore, the aim of the study was to explore the correlation between the markers of systemic and periodontal inflammation as assessed by CRP and the PSGS in participants with periodontitis.

Materials and methods

The present study enrolled 85 systemically healthy participants with periodontitis. PSGS and CRP levels were measured in each participant. Descriptive and inferential statistics were applied for analysis.

Results

The PSGS scores ranged from 24 to 213, and the CRP levels ranged from 0.5 to 3.23 mg/l. This shows a positive correlation between the periodontal scale and CRP. A nonsignificant (p-value > 0.05) correlation exists between age and CRP score, and a significant association was seen between gender and severity of periodontitis with p-value < 0.02. A highly significant association between gender and CRP score was found with p-value < 0.001.

Conclusion

The level of serum CRP dramatically increased with the severity of periodontitis. The results of this study point to a highly significant correlation between markers of systemic and periodontal inflammation, as well as a strength of association between the two markers.

## Introduction

The disease of the gingiva affects about 20-50% of the population in all age groups globally, making it a civic health concern [1]. The oral cavity is a documented potential source of systemic infection as microorganisms enter the blood through the periodontium and cause chronic inflammation.

One of the most common acute phase proteins is C-reactive protein (CRP), which increases at sites of inflammation. CRP is a pentamer that was first discovered by Tillet and Francis in 1930 [2]. Owing to the inflammatory changes in the periodontium, patients with periodontitis may have altered molecular and cellular components in their peripheral blood, and this low-grade systemic inflammation that is fueled by regional inflammatory mediators results in the production of CRP [3,4].

Many indexes are used to measure periodontal destruction, such as the community periodontal index, extent and severity index, periodontal screening and recording index, etc., but none of them have been established as a gold standard. The periodontitis severity grading scale (PSGS) was developed by the authors to measure the intensity of periodontal inflammation. Specifically, the PSGS was used to measure the severity and extent of inflammation in periodontal tissues during routine clinical periodontal examination in each patient. Compared to existing indices, the PSGS is a four-parameter comprehensive scale for periodontal disease that provides an accurate depiction of periodontal inflammation. The new scale is simple and easy and can be measured within five minutes in a clinical setting. The scale is validated, but research has not yet been published. Six locations per tooth per patient with probing pocket depths of 4 mm or greater, as well as the number of teeth with bleeding on probing, evident suppuration, and furcation lesions, resulted in a final aggregate score reflecting the patient's periodontal disease condition. The present study assessed the role of CRP and PSGS as systemic and periodontal markers, respectively. It evaluated the stages of periodontitis and its correlation to CRP levels as an inflammatory marker and subsequently evaluated the patients' risk for cardiovascular disease.

## Materials and methods

The present observational pilot study was conducted as part of PhD research. Systemically healthy patients with periodontitis aged 20-60 years and fulfilling the inclusion criteria were recruited from the Outpatient Department of Periodontology. Participants with a medical history, smoking habit, or history of antibiotic therapy in the past three months after having undergone periodontal treatment were excluded to avoid confounding the results. The study commenced after institutional ethics committee approval was acquired with IRB number: SVIEC/ON/DENT/PhD/18004. Written informed consent was obtained from each participant after the purpose and significance of the study were explained. The total sample size for the study was calculated according to a study conducted by Malikabood et al. [5], where 85 systematically healthy participants with periodontitis of Stages I, II, and III and Grades A, B, C, and 8 were considered for the study.

The PSGS is an aggregate full-mouth scale to determine periodontal inflammation. The parameter used in the scale to measure gingival inflammation was the presence or absence of bleeding on probing per tooth. The parameters used to measure periodontitis included 1) probing depth, 3) the presence or absence of suppuration, and 4) the presence or absence of furcation lesion. A probing depth of 4 mm or more was taken into consideration in six sites per tooth (mesio-buccal, buccal, distobuccal, mesio-lingual, lingual, and distolingual). A pressure-sensitive probe (Axe pressure sensitive probe, Bluedent India, Tamil Nadu, India) was used to measure the depth of the pocket, and Naber's probe PQ2N (GDC Fine Crafted Dental Pvt. Ltd. Hoshiarpur, Punjab, India) was used for the presence of furcation lesion. The severity of periodontitis was measured according to the 2017 Classification of Periodontal Disease and Peri-implantitis [4]. Demographic data, along with the stage of periodontitis, were recorded in proforma. The authors have applied for a copyright for the PSGS in India.

Venous blood samples were obtained from the central veins in the antecubital fossa. Three milliliters of blood were drawn using a 5-cc disposable syringe and 23-gauge needles. The blood was allowed to clot and then centrifuged at 3,000 revolutions per minute (rpm) for five minutes, and the serum was separated to estimate the CRP levels using a latex-enhanced turbidimetric immunoassay (Mispa-i2 Specific Protein Analyzer, Agappe Diagnostics Ltd, Kerala, India). The CRP levels have a long-lasting prognostic value and are considered as a biomarker for cardiovascular disease. Low-, moderate-, and high-risk categories of cardiovascular disease are represented by CRP values of 1, 1-3, and >3 mg/L [6].

The data were subjected to statistical analysis with the Statistical Package for Social Sciences (SPSS software version 21.0, SPSS Inc, IBM Corp., New York, USA). Descriptive and inferential statistics were used, and the correlation between CRP and PSGS was analyzed using the Spearman rank correlation coefficient. Cramér's V test was used to check the strength of the association between variables. The level of significance was set at p < 0.05.

## Results

A total of 85 patients, with a mean age of 43.27 ± 11.94 years, 16 (18.8%) males and 69 (81.2%) females, with ages ranging from 20 to 60, were enrolled in the study. The periodontal status recorded using the PSGS ranged from 20 to 213, and the CRP levels ranged from 0.5 to 3.23 mg/l.

**Figure 1:**
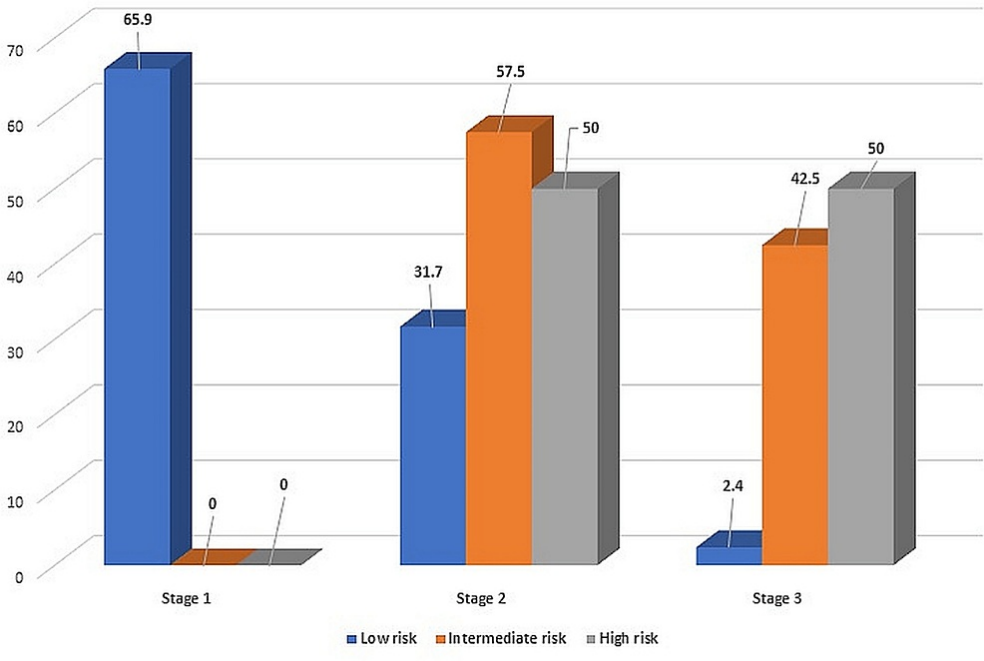
Association risk of cardiovascular disease with the severity of periodontitis

To check the normality of the PSGS and CRP data, we used the Kolmogorov-Smirnov test. This test indicated that the data for the PSGS were not normally distributed (p-value = 0.04, null hypothesis rejected), and the data for the CRP were not normally distributed (p-value = 0.01, null hypothesis rejected). Therefore, the non-parametric test (Spearman's rho correlation) was used to find out if there was a correlation between the PSGS and CRP score (Table 1, Figure 1).

**Table 1:** Correlation between the PSGS and CRP scores CRP: C-reactive protein PSGS: Periodontitis severity grading scale

Variables			PSGS	CRP
Spearman's rho	PSGS	Correlation coefficient	1.000	0.793
		Sig. (2-tailed)	.	0.000
		N	85	85
	CRP	Correlation coefficient	0.793	1.000
		Sig. (2-tailed)	0.000	.
		N	85	85

A total of 85 patients, with a mean age of 43.27 ± 11.94 years, 16 (18.8%) males and 69 (81.2%) females, were enrolled in the study. The periodontal status recorded using the PSGS ranged from 20 to 213, and the CRP levels ranged from 0.5 to 3.23 mg/l (Table 2).

**Table 2:** Association between the severity of periodontitis and C-reactive protein (CRP) mg/L: Milligrams per liter N: Number of patients %: Percentage

Risk of cardiovascular disease, Severity of periodontitis	Low risk (≤ 1.00 mg/L) N (%)	Intermediate risk (1.99 mg/L) N (%)	Intermediate risk (2.00-2.99 mg/L) N (%)	High risk (≥ 3.00 mg/L) N (%)	Total
Stage 1	27 (65.9%)	0	0	0	27 (100.0%)
Stage 2	13 (31.7%)	15 (53.6%)	8 (66.7%)	2 (50.0%)	38 (100.0%)
Stage 3	1 (2.4%)	13 (4.4%)	4 (33.3%)	2 (50.0%)	20 (100.0%)
Total	41 (100%)	28 (100.0%)	12 (100.0%)	4 (100.0%)	85 (100.0%)
Chi-square value = 48.09, d.f = 6 , p-value < 0.001					

A positive 0.793 (79.3%) and highly significant correlation (p-value < 0.01) was observed between the PSGS and CRP. We found a negative 0.04 (4%) and nonsignificant (p-value > 0.05) correlation between age and CRP score. The strength of association was obtained using Cramér's V test, which gave a value of 0.531 (53.1%) with p-value < 0.001.

A highly significant association between gender and CRP score was found with the strength of association of 0.467(46.7%) and p-value < 0.001 (Table 3).

**Table 3:** Association between gender and CRP levels CRP: C-reactive protein mg/L: Milligrams per liter N: Number of patients %: Percentage

Gender versus CRP levels	≤ 1.00 mg/L N (%)	1.01-1.99 mg/L N (%)	2.00-2.99 mg/L N (%)	≥ 3.00 mg/L N (%)	Total N (%)
Male	9 (22.0%)	6 (21.4%)	8 (66.7%)	4 (100.0%)	27 (100.0%)
Female	32 (78.0%)	22 (78.6%)	4(33.3%)	0	58 (100.0%)
Total	41 (100.0%)	28 (100.0%)	12 (100%)	4 (100.0%)	85 (100.0%)
Chi-square value = 18.53, d.f = 3 , p-value < 0.0001					

There was a highly significant correlation established between gender and severity of periodontitis, with a strong association of 0.314. The correlation was also ascertained between gender and CRP, with a strong association of 0.467. The p-value for the correlation was 0.01 where the severity and gender were showing a positive relationship (Table 4).

**Table 4:** Association between gender and severity of periodontitis N: Number of patients %: Percentage

Gender versus severity of periodontitis	Stage 1 N (%)	Stage 2 N (%)	Stage 3 N (%)
Male	3 (11.1%)	17 (44.7%)	7 (35.0%)
Female	24 (88.9%)	21 (55.3%)	13 (65.0%)
Total	27 (100.0%)	38 (100.0%)	20 (100.0%)
Chi-square value = 8.32, d.f = 2 , p-value = 0.01			

## Discussion

The PSGS used in the present study was developed by the researchers and included parameters such as bleeding on probing, probing pocket depth, evident suppuration, and involvement of furcation. It considers not only the destruction of the periodontal tissue but also the gingival aspect. Owing to the inflammatory changes in the periodontium, patients with periodontitis may have altered molecular and cellular components in their peripheral blood. As an acute-phase protein, CRP is a biomarker for systemic inflammation. It is synthesized in the liver when there is tissue damage or inflammation and can also be produced by arterial tissue [7].

In a study conducted by Bolla et al., CRP was investigated in healthy patients and those with generalized aggressive (GAP) and chronic periodontitis (CP). The CRP levels were greater in the GAP and CP groups compared with the healthy controls. CRP was found to be higher in CP cases than in GAP cases, although this was statistically nonsignificant. Over 50% of cases (GAP and CP) exhibited CRP levels of more than 3 mg/l, a finding which is similar to the present study [8]. In their study, Glurich et al. reported elevated CRP levels when bleeding was present on probing and CAL was greater than or equivalent to 4 mm [9]. Noack et al. showed an increase in CRP in patients who showed periodontal destruction equal to or greater than 3 mm [10], which is similar to the present study, where probing pocket depth was more than or equal to 4 mm. For mild, moderate, and severe chronic periodontitis, the mean CRP values were 1.0, 2.4, and 4.1 mg/l, respectively. The relationship between serum CRP levels and periodontitis severity was statistically significant (p = 0.006), which was similar to the findings of the present investigation [11]. CRP can be an objective indicator of disease activity in patients with periodontal disease. A further strength of association was studied, which showed a strong association (0.531) between the severity of periodontitis and CRP. CRP increases with age, but in our study, there was no correlation between age and CRP level. There was a highly significant correlation established between gender and severity of periodontitis, with a strong association of 0.314. The correlation was also ascertained between gender and CRP, with a strong association of 0.467.

CRP can be a biomarker for coronary risk assessment, where scores greater than the cut-point of 3 mg/l indicated subjects at high risk [4-11]. Shah et al. investigated the levels of high sensitive CRP (hs-CRP) using a periodontal destruction dental index score for 20 individuals with chronic periodontitis [4]. Their results showed that, when the dental index score was 95 ± 47.77 and the hs-CRP was 3.29 ± 4.66 mg/l, the patients were at intermediate to high risk of cardiovascular disease, whereas in the present study, most of the patients were in the medium-risk category [12].

These results substantiate that periodontal disease is associated with increased levels of CRP and that increased CRP levels associated with periodontitis may contribute to systemic vascular inflammation, atheroma development, and an increased risk of pre-existing cardiovascular conditions. A CRP level of more than 10 mg/L is associated with an increased chance of getting life-threatening cardiovascular disease within 10 years [13,14]. As reported in a systematic review and meta-analysis by Singh et al., who stated that CRP, can be utilized as a predictor for cardiovascular disease [15], Glurich et al. found that CRP levels increased two times when either periodontitis or cardiovascular disease was present and three times when both conditions were present [9].

The study has some limitations. To further evaluate the relationship between periodontitis and serum levels of CRP, however, more research is required, particularly interventional and longitudinal studies that pay close attention to confounding variables.

## Conclusions

The PSGS depicts periodontal pathology by examining the number of sites of diseased periodontal areas. It is a valid measure of periodontal inflammation. The current system of grading and severity is extensive and time taking. The new scale gives an encompassing view of the periodontal inflammatory burden, rather than a single parameter, with which the serum level of CRP can be directly measured. High CRP levels indicate ongoing tissue injury, and they could be an indicator of an increase in local disease severity. To conclude, the results of the study demonstrate a correlation between markers of systemic and periodontal inflammation.
